# Commentary: Depression score and body mass index mediate the association between dietary vitamin C intake and female infertility: a study based on NHANES 2013–2018

**DOI:** 10.3389/fnut.2026.1717289

**Published:** 2026-02-19

**Authors:** Nan-ren Sun, Zhidong Zhou, Zhimin Tan

**Affiliations:** 1 Shandong University of Traditional Chinese Medicine, Jinan, Shandong, China; 2 Lin'an District Hospital of Traditional Chinese Medicine, Hangzhou, Zhejiang, China; 3 Department of Otorhinolaryngology, The Affiliated Hospital, Shandong University of Traditional Chinese Medicine, Jinan, Shandong, China

**Keywords:** vitamin C, infertility, NHANES, female reproductive health, oxidative stress

## 1 Introduction

The article “Depression score and body mass index mediate the association between dietary vitamin C intake and female infertility: a study based on NHANES 2013–2018” (1) by Zheng et al. provides important new insights into the role of vitamin C in reproductive health. Infertility has become a major global public health challenge, and nutritional interventions represent a relatively affordable, feasible, and scalable strategy. By focusing on vitamin C, a common and modifiable dietary factor, the authors not only expand our understanding of the etiology of infertility but also provide valuable insights for clinical prevention and public health strategies. Methodologically, the article demonstrates remarkable strengths. The authors performed multivariable regression analyses, and further conducted sensitivity analyses, subgroup analyses, mediation analyses, and explored potential non-linear relationships. These complementary approaches substantially enhance the robustness and credibility of the findings. Such a comprehensive and systematic analytical framework provides an excellent model for future research in nutritional epidemiology.

## 2 Subsections relevant for the subject

### 2.1 Source of vitamin C intake

The study did not distinguish between food-derived and supplement-derived vitamin C, which represents an important area for improvement. Ample evidence indicates that oxidative stress (OS) plays a critical role in female reproductive health (2). Vitamin C from natural foods is usually accompanied by flavonoids, polyphenols, and other bioactive plant compounds (3). These compounds not only enhance antioxidant and cytoprotective effects but may also improve the ovarian microenvironment and reduce OS, thereby contributing to the maintenance of reproductive function. Such synergistic effects may play an important role in the prevention and management of infertility, whereas supplementation alone may not fully reproduce these benefits (4). Moreover, high-dose supplements are often limited by intestinal absorption saturation and renal excretion, which may alter their physiological effects (5). In fact, NHANES data explicitly distinguish nutrient sources from foods/beverages and from dietary supplements, and researchers can easily merge these datasets by respondent identifier. While focusing on the potential role of vitamin C in reproductive health is well-justified, incorporating source-specific analyses would allow a more accurate evaluation of vitamin C's independent role in infertility, while avoiding potential confounding between dietary habits and supplement use. This would further improve the scientific rigor and credibility of the study's conclusions.

### 2.2 Application of survey weights

The authors did not clearly indicate whether appropriate weighting was applied to account for the complex sampling design of NHANES. Given that NHANES is a stratified, multistage survey, the use of sampling weights, clustering, and stratification adjustments is strongly recommended, particularly when combining multiple cycles (6). Without weighting, findings may primarily reflect the study sample rather than the U.S. population, thus limiting generalizability. It is possible that this omission relates to sample size constraints or analytic simplicity; however, without direct access to the data processing steps, we cannot be certain. It would be helpful if the authors could clarify this point in their methodology, and future studies may benefit from fully incorporating weighting procedures to further strengthen robustness and public health relevance.

### 2.3 Multiple comparisons and statistical power

The study involved multiple comparisons, including subgroup analyses, non-linear dose–response modeling, and parallel analyses of several micronutrients, but did not implement formal multiple testing corrections (e.g., Bonferroni or false discovery rate), which may increase the risk of type I error (7). Furthermore, no power analysis was reported. Our *post-hoc* calculation indicates that in the primary analysis (*n* = 2,381; fertile women = 2,100, infertile women = 281), the statistical power to detect the observed effect size (OR ≈ 0.61) at a two-sided α = 0.05 was approximately 64%. In subgroup analyses, the power was even lower (≈43% in women aged 18–34 years, ≈30% in women aged 35–44 years). This likely accounts for the instability observed in the subgroup analyses, where effect estimates were directionally consistent but lacked statistical significance due to wide confidence intervals.

## 3 Discussion

In summary, this is an excellent and valuable study that advances our understanding of the relationship between micronutrient intake and infertility. At the same time, the noted limitations highlight opportunities for future research, including the use of larger cohorts, more precise exposure assessment, appropriate multiple testing corrections, and analyses that incorporate dietary patterns. Addressing these issues will further confirm and expand the important contributions of this work.
